# Transitions in Adherence Trajectories From Intervention to Maintenance of a Falls Prevention Exercise Program

**DOI:** 10.1093/geroni/igab046.1732

**Published:** 2021-12-17

**Authors:** Pildoo Sung, May-Ling June Lee, Kok Yang Tan, Rahul Malhotra, Angelique Chan

**Affiliations:** Duke-NUS Medical School, Singapore, Singapore

## Abstract

The successful implementation of a falls prevention exercise program for older adults hinges on self-maintenance after active intervention. However, little is known about the pattern of adherence from the intervention to the maintenance phase of such programs, and the factors influencing adherence. We investigate transitions in exercise adherence trajectories from the active intervention to the maintenance phase of a falls prevention exercise program in Singapore, and whether exercise self-efficacy is associated with adherence in the maintenance phase. We analyze data of 143 older adults who participated in a 12-week, group-based falls prevention exercise program, followed by a 6-month maintenance phase, in 2018-2019. Sequential process latent class growth modeling identifies the distinct exercise adherence trajectories in the active intervention and the maintenance phase separately and their transition patterns. Multivariable regression examines whether baseline and change in self-efficacy during the active intervention predict adherence during the maintenance phase. The analysis reveals three exercise adherence trajectories— adherent (40% of participants), intermittent (38%), and disengaged (22%)—in the active intervention phase, and two trajectories—adherent (33%) and disengaged (67%)—in the maintenance phase. Those adherent in the maintenance phase comprise participants who were adherent (42%) or intermittent (58%) in the active intervention phase. Baseline and increase in exercise self-efficacy during the active intervention are positively associated with adherence in the maintenance phase. The findings capture the heterogeneity in exercise adherence patterns within and across the active intervention and maintenance phases of falls prevention exercise program, and the importance of exercise self-efficacy in continued adherence to exercise.

